# The risk of venous thromboembolism in adult patients with diffuse glioma: a nationwide population-based study

**DOI:** 10.2340/1651-226X.2024.40137

**Published:** 2024-11-14

**Authors:** Frederik R. Hovman, Frantz R. Poulsen, Steinbjørn Hansen, Rikke H. Dahlrot

**Affiliations:** a Department of Oncology, Odense University Hospital, Odense, Denmark; b Department of Clinical Research, University of Southern Denmark, Odense, Denmark; c Department of Neurosurgery, Odense University Hospital, Odense, Denmark; d Department of Clinical Research, and BRIDGE (Brain Research – Inter Disciplinary Guided Excellence), University of Southern Denmark, Odense, Denmark

**Keywords:** Cancer-associated thrombosis, brain tumor, glioblastoma, epidemiology, risk factors

## Abstract

**Background and purpose:**

Venous thromboembolism (VTE) is a cause of increased morbidity and risk of death. Studies report VTE in up to 30% of glioma patients but the results vary. The VTE risk is relevant when evaluating prophylaxis to avoid unnecessary bleeding or overdiagnosis. This study examines the VTE incidence in patients with glioma WHO grade 2–4, and when VTE occurred, risk factors, and overall survival (OS) for patients with WHO grade 4.

**Materials and methods:**

In total 3,630 patients with WHO grade 2 (*n* = 230), grade 3 (*n* = 317), and grade 4 (*n* = 3,083) gliomas from 2010 to 2018 were identified using the Danish Neuro-Oncology Registry. VTE diagnoses and time of death were obtained from Statistics Denmark.

**Results and interpretation:**

The VTE incidence was 5.2, 6.3, and 6.8% in patients with WHO grade 2, 3, and 4 gliomas, respectively. The VTE incidence rate was highest during the first 3 months after the diagnosis with 53 events. Increasing age (HR 1.03, 95%CI 1.01–1.04), male sex (HR 1.47, 95%CI 1.09–1.99), poor performance status (HR 1.57, 95%CI 1.10–2.25), and post-operative long-course radiochemotherapy (HR 2.10, 95%CI 1.19–3.72) were predictors of VTE in patients with glioma WHO grade 4. There was no difference in OS comparing patients having VTE to those without (*p* = 0.068). In conclusion, patients with glioma WHO grade 2–4 were at high risk of VTE, especially the first 3 months after diagnosis. Increasing age, male sex, poor performance status, and long-course radiochemotherapy were associated with increased risk of VTE in patients with glioma WHO grade 4.

## Background

Venous thromboembolism (VTE) is a frequent complication among patients with cancer, who carry a risk up to nine times that of the general population [1]. In patients with primary brain tumors, the incidence of VTE is often reported to be 20–30% [2, 3] although previous studies report incidences ranging from 3.5 to 60% [4, 5]. In contrast, prophylactic anticoagulant therapy may increase these patients’ risk of intracranial bleeding [6, 7].

The mechanism behind cancer-associated thrombosis is not fully understood, but high tumor grade, poor performance status (PS), isocitrate dehydrogenase (IDH) wildtype, small extent of surgery, chemotherapy, age, sex, and comorbidity have been associated with VTE [2, 6, 8].

The risk of VTE is increased during the postoperative period [3, 9–17], but studies suggest that the risk remains high throughout the course of the disease [3, 9–17]. Studies report that VTE does not affect survival [12, 15, 17, 18], whereas others report increased mortality in patients with VTE [9, 14].

Previous studies investigating VTE in glioma patients differ methodologically for example regarding tumor grade, length of follow-up, inclusion of asymptomatic events, and screening. Recent changes in the WHO glioma classification also reduce the generalizability for patients today as most larger studies are based on populations from 1993 to 2011 [9, 4,18–20].

The objective of this national population-based study was to determine the incidence of VTE in Danish patients diagnosed with glioma WHO grade 2–4 during 2010–2018. For patients with glioma WHO grade 4, the time of VTE, risk factors for VTE, and overall survival (OS) were evaluated.

## Methods

### Inclusion and outcomes

Patients with primary glioma during 1 January 2010–31 December 2018 were identified and characterized using The Danish Neuro-Oncology Registry (DNOR), a national clinical cancer database with high completeness (92%) [21].

The Danish National Patient Registry (DNPR) and Statistics Denmark provided time of death, diagnoses, and admission dates. All databases were linked using the Danish civil registration (CPR) number, which is unique for each person in Denmark. The CPR number is mandatory in all contacts with the Danish health care system resulting in close-to-complete follow-up [22]. The validity of VTE diagnoses from DNPR has been validated with positive predictive values of 75–90% for in- and outpatient contacts [23].

The original tumor grade was used, yet available IDH wildtype tumors were reclassified as grade 4 according to the WHO 2021 classification [24].

VTE was defined as the first diagnosis of deep vein thrombosis or pulmonary embolism. The recommended perioperative prophylaxis of VTE in patients with glioma included compression stockings and low molecular weight heparin until the patient was mobilized [25]. If VTE occurred ≤3 months before the primary glioma diagnosis, it was defined as related to the glioma.

### Statistical analysis

Patient characteristics were described using descriptive statistics, Wilcoxon’s rank sum test (continuous variables), and χ^2^ test or Fisher’s exact.

The incidence of VTE was described as the crude incidence and the cumulative incidence function adjusted for mortality.

The time of VTE was described as the frequency and incidence rate of VTE during time intervals before and after the glioma diagnosis for patients with glioma WHO grade 4.

Cox regression for VTE was performed for patients with glioma WHO grade 4 using pre-selected covariates. All assumptions were tested and fulfilled.

OS is defined as time from the primary surgery until death or censoring (December 31st 2018) and was illustrated using the Kaplan–Meier plot.

Analyses were two-tailed and conducted in STATA 17.0 with 5% significance level. To avoid publishing microdata, cells with <3 observations were censored in accordance with regulations from Statistics Denmark [26].

### Ethics

The study was approved by the Regional Council of Southern Denmark (J.nr. 22/10281) and the Danish Data Protection Agency (J.nr: 22/13259).

## Results

### Population

**Figure 1:**
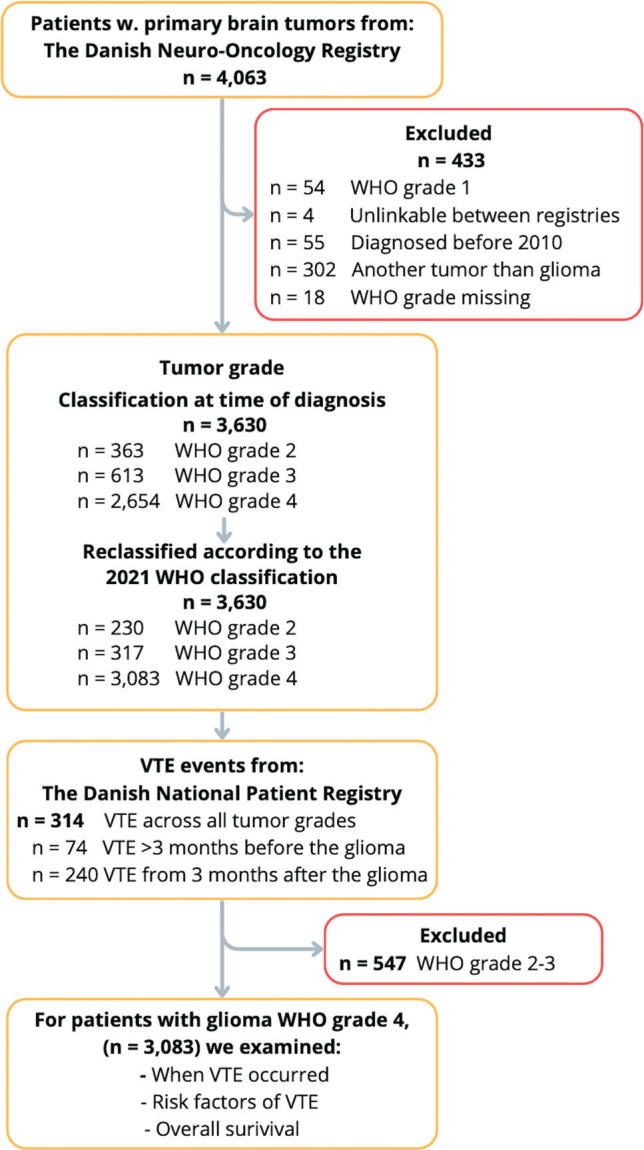
Flowchart showing the inclusion of patients with glioma WHO grade 2–4 using national registries. VTE: venous thromboembolism; CPR: civil registration number (unique to all Danish citizens).

In total 4,063 patients were identified, of whom 433 were excluded (Figure 1). Using the original WHO classification, 363 patients had WHO grade 2, 613 patients WHO grade 3, and 2,654 patients had glioma WHO grade 4. After reclassification, the number of patients with glioma WHO grade 4 increased to 3,083.

### Patient characteristics

The median age was 63 years (range 18–95), and 59% were male. Most patients (76%) had Charlson Comorbidity Index (CCI) of 1–2 and 85% had glioma WHO grade 4. As the primary surgery 32% of patients had a biopsy, 34% had subtotal resection, and 34% had maximum safe resection. VTE occurred in 314 patients, 37% had deep vein thrombosis and 63% had pulmonary embolism.

The type of VTE was distributed as 57% PE and 43% DVT for WHO grade 2 patients, 42% PE and 58% DVT for WHO grade 3, and 65% PE and 35% DVT for patients with WHO grade 4.

Patients with glioma WHO grade 2 and 3 were generally younger without substantial comorbidity (Supplementary Table 1). A total of 60% patients with glioma WHO grade 4 were male, 85% had no comorbidity or low CCI, and 49% received long-course radiochemotherapy (Table 1) (See Supplementary Table 2 for supplementary characteristics of patients with glioma WHO grade 4). Patients with glioma WHO grade 4 and VTE were more likely to be male than female (*p* = 0.005). In the patients with VTE, 67% received long-course radiochemotherapy compared to 48% in the group without VTE (Table 1).

**Table 1:** Characteristics of patients with and without VTE for patients with glioma WHO grade 4.

	Total	No VTE	VTE	*p*
	*n* = 3,083	*n* = 2,875	*n* = 208	
Age (median, range)	65 (18–95)	65 (18–95)	65 (24–85)	0.84
Sex				**0.005**
Male	1,848 (60%)	1,704 (59%)	144 (69%)	
Female	1,235 (40%)	1,171 (41%)	64 (31%)	
Focality				0.97
Unifocal	2,492 (81%)	2,325 (81%)	167 (80%)	
Multifocal	470 (15%)	437 (15%)	33 (16%)	
Missing	121 (4%)	113 (4%)	8 (4%)	
Charlson Comorbidity Index				0.54
0	209 (7%)	191 (7%)	18 (9%)	
1–2	2,422 (78%)	2,262 (78%)	160 (77%)	
≥ 3	452 (15%)	422 (15%)	30 (14%)	
IDH1 & IDH2 status				0.76
Wild type	2,307 (75%)	2,147 (75%)	159 (77%)	
Mutated	36 (1%)	34 (1%)	3 (1%)	
Missing	740 (24%)	694 (24%)	46 (22%)	
MGMT promoter status				0.94
Unmethylated	1,672 (54%)	1,558 (54%)	114 (55%)	
Methylated	668 (22%)	625 (22%)	43 (21%)	
Missing	743 (24%)	692 (24%)	51 (25%)	
Extent of surgery				0.35
Biopsy	1,001 (32%)	945 (33%)	56 (27%)	
Subtotal resection	1,053 (34%)	975 (34%)	78 (38%)	
Radical resection	1,028 (33%)	954 (33%)	74 (36%)	
Performance status before surgery				0.81
0	982 (32%)	912 (32%)	70 (34%)	
1	1,071 (35%)	999 (35%)	72 (35%)	
≥ 2	1,030 (33%)	964 (34%)	66 (32%)	
Post-operative radiochemotherapy				**< 0.001**
None	530 (17%)	516 (18%)	14 (7%)	
Short-course	911 (30%)	863 (30%)	48 (23%)	
Long-course	1,513 (49%)	1,374 (48%)	139 (67%)	
Missing	129 (4%)	122 (4%)	7 (3%)	
Overall survival, months (median, range)	10.7 (0.1–107.5)	10.3 (0.1–107.5)	15.0 (0.4–72.6)	0.068

Characteristics of patients with glioma WHO grade 4. Continuous measures are presented as median (range) and are compared using the Wilcoxon rank sum test. Categorical measures are presented as *n* (%) and are compared using the χ^2^-squared test or Fisher’s exact test if <5 values were observed.

Post-operative radiochemotherapy included patients who received < 59.4 Gy of radiation therapy with or without chemotherapy as ‘Short-course’ and patients who received ≥ 59.4 Gy plus concomitant and adjuvant temozolomide as ‘Long-course’.

Values that are significant at a 5% significance level are highlighted in bold text.

VTE: Venous thromboembolism; IDH: Isocitrate dehydrogenase; MGMT: O^6^-methylguanine-DNA methyltransferase.

**Table 2:** Multivariate Cox regression for venous thromboembolism in patients with glioma WHO grade 4.

	HR	95% CI		*p*
Age at diagnosis (years)	1.03	1.01–1.04		**< 0.00**
Sex				
Female	1.00			
Male	1.47	1.09–1.99		**0.01**
Extent of surgery				
Biopsy	1.00			
Subtotal resection	0.93	0.65–1.34		0.70
Radical resection	0.85	0.59–1.23		0.40
Performance status				
0	1.00			
1	1.16	0.82–1.64		0.4
≥ 2	1.57	1.10–2.25		**0.01**
Charlson Comorbidity Index				
0	1.00			
1–2	0.75	0.44–1.28		0.29
≥ 3	0.87	0.46–1.63		0.66
Post-operative radiochemotherapy				
None	1.00			
Short-course	1.35	0.74–2.47	0.33	
Long-course	2.10	1.19–3.72	**0.01**	

Results of multivariate Cox proportional hazards regression for venous thromboembolism in 208 patients with venous thromboembolism and glioma WHO grade 4.

Postoperative treatment included patients who received < 59.4 Gy of radiation therapy with or without chemotherapy as ‘Short-course’ and patients who received ≥ 59.4 Gy plus concomitant and adjuvant temozolomide as ‘Long-course’.

HR: hazard rate.

For patients with glioma WHO grade 2, VTE was correlated with CCI, type of surgery, and postoperative radiotherapy. For patients with glioma WHO grade 3, VTE was correlated with increasing age (Supplementary Table 1).

### Incidence of VTE

The crude incidence of VTE was 5.8% (21/363) for WHO grade 2, 5.5% (34/613) for WHO grade 3, and 7.0% (185/2,645) for glioma WHO grade 4 using the WHO classification at the time of diagnosis. After reclassification, VTE occurred in 5.2% (12/230) of patients with WHO grade 2, 6.3% (20/317) with WHO grade 3, and 6.8% (208/3,083) of patients with glioma WHO grade 4.

**Figure 2:**
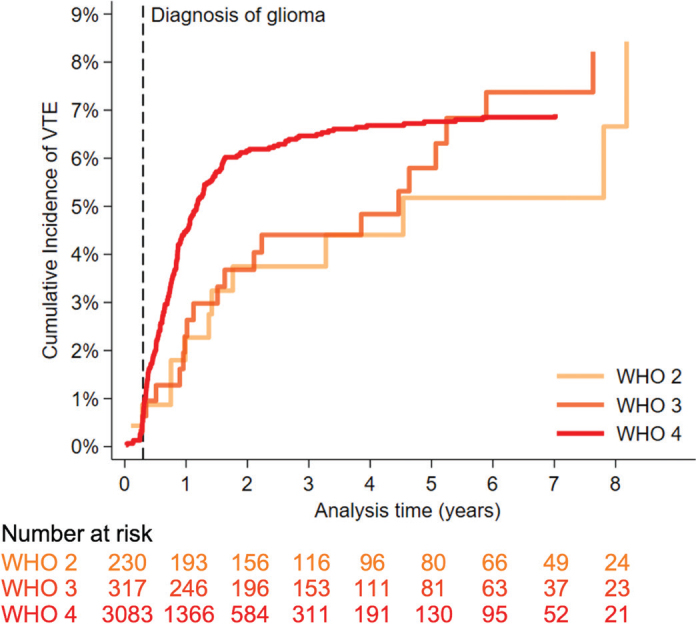
Cumulative incidence of venous thromboembolism adjusted for mortality in 3,630 patients diagnosed with glioma WHO grade 2, 3, or 4. The risk table shows the number of patients who were still alive and had not experienced VTE.

The adjusted cumulative VTE incidence was 8.4% (95%CI 4.1–14.8) for patients with WHO grade 2, 8.2% (95%CI 5.1–12.3) for patients with grade 3, and 6.9% (95%CI 6.1–7.9) for patients with glioma WHO grade 4 (Figure 2).

### VTE in patients with glioma WHO grade 4

Seventy-four patients (2.3%) had VTE before the glioma diagnosis, and six (8%) events occurred within the last 3 months before the glioma diagnosis (Supplementary Table 3). After the glioma diagnosis, VTE occurred in 200 patients, whereof 26.5% (*n* = 53) were within the first 3 months, 48% (*n* = 95) within 6 months, 76% (*n* = 151) within 12 months, and 91% (*n* = 181) were within the first 24 months after the diagnosis.

**Figure 3:**
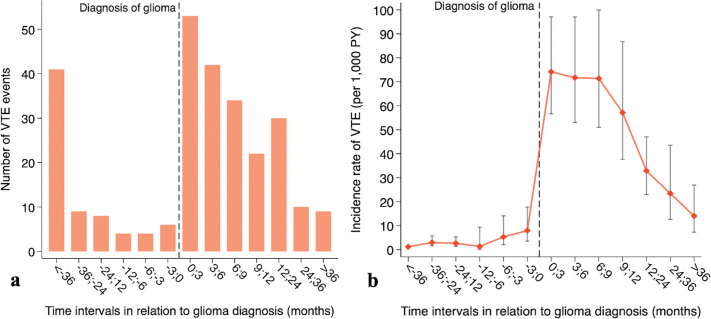
(A) Bar plot depicting the distribution of 274 VTE events for 3,083 patients with glioma WHO grade 4 during time intervals of varying lengths. (B) Graph illustrating the corresponding incidence rates of VTE and 95% confidence intervals during the same time intervals. VTE: Venous thromboembolism; PY: person-years.

**Figure 4:**
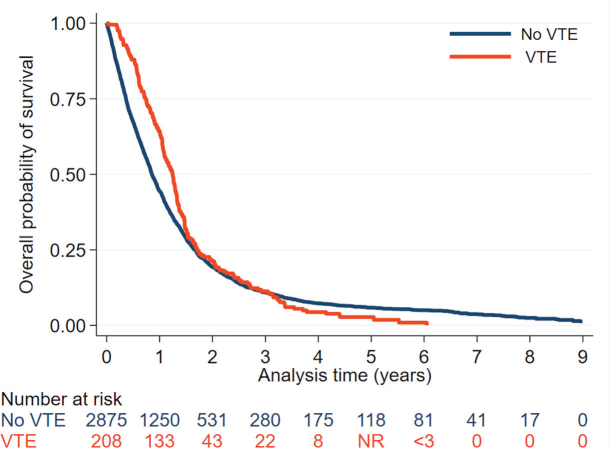
Kaplan–Meier plot depicting overall survival after pathologically verified diagnosis of glioma WHO grade 4 for patients with and without VTE from 3 months before the glioma diagnosis. 1-year OS was 63.9% in the group with VTE and 44.5% in the non-VTE group. VTE: venous thromboembolism; < 3, less than 3 observations; NR: not reported to avoid publishing of microdata.

The overall incidence rate of VTE after the glioma diagnosis was 48 VTE events per 1,000 person-years (95%CI 42–56), which peaked during the first 3 months at a rate of 74 VTE events per 1,000 person-years (95%CI 57–97) (Figure 3, Supplementary Table 4). Using Cox regression, increasing age, male sex, PS ≥ 2, and long-course radiochemotherapy were identified as risk factors for VTE (Table 2). There was no statistical difference in OS between the group with VTE compared to the non-VTE group (*p* = 0.068) (Table 1, Figure 4).

## Discussion

We report a high incidence of VTE in glioma patients, but not as high as previous studies [4, 10–13, 15, 18, 19, 27–29]. This may be due to heterogeneous methods like use of registry-based studies [9, 20, 30] compared to medical chart reviews [13, 18, 28] or prospective studies [10, 11, 15, 19, 27, 29]. Further, studies might identify both symptomatic and asymptomatic VTE if they use screening [5, 12, 16, 29] or VTE risk-assessment tools [31], but the clinical use is still undecided, as it may result in unnecessary worry and treatment [31]. The national guidelines did not encourage screening during the current study [25], leading us to assume that most events were symptomatic, and that screening could have identified more events. Further, use of antithrombotic agents might reduce the risk of VTE. The impact of this is difficult to assess, as the description of VTE prophylaxis is unclear in other studies [10, 12, 13, 18]. The use of antithrombotic agents in our population is unknown, but the national guidelines did not recommend routine use of anticoagulants, except 24 h before surgery [25]. VTE diagnoses was obtained using close-to-complete follow-up national databases. However, studies validating VTE diagnoses from DNPR have found lower positive predictive values from emergency departments compared to in- and outpatient contacts. This might result in underestimation of the VTE incidence in the current study [23].

For patients with glioma WHO grade 4, most VTE events occurred during the first 12 months after the glioma diagnosis which is consistent with previous studies [4, 9–17].

We observed a slightly increased VTE incidence before the glioma diagnosis. This should be interpreted with caution as it is based on just a few events, yet it is consistent with the results of Mulder et al. [1], who reported an increased risk of VTE (HR 3.6) 6 months before diagnosis.

Like previous studies [9, 11,12, 14, 27, 28, 30], we identified increasing age, male sex, PS ≥2, and post-operative long-course radiochemotherapy as risk factors for VTE, but we did not confirm the extent of surgery or comorbidity as risk factors. Comorbidity has been associated with an increased risk of VTE in cancer patients [6], but to our knowledge, only Semrad et al. [9] have reported on glioma patients. Semrad et al. [9] used the Elixhauser comorbidity index instead of CCI, which might explain why their results differ from ours, as this index is thought to have a superior prognostic value [32].

There was no difference in OS between patients with and without VTE, which is consistent with previous studies [12, 15, 17, 18, 28]. Surprisingly, 1-year OS was higher for patients with VTE compared to those without, which we speculate might be partly due to underdiagnosis of non-symptomatic VTE and that lack of anticoagulant treatment could have affected OS. Due to the nature of this study, we cannot assume a causal relationship.

In conclusion, we report a high risk of VTE in patients with glioma but not as high as previous studies. The risk of VTE was highest within the first 12 months after the glioma diagnosis. Prospective studies examining VTE screening and its effect on outcomes for patients with glioma are needed.

## Supplementary Material

The risk of venous thromboembolism in adult patients with diffuse glioma: a nationwide population-based study

## Data Availability

Data obtained from the registries is not allowed to be made publicly available according to Statistics Denmark [26].
