# Supplementary material for: The risk of venous thromboembolism in adult patients with diffuse glioma: a nationwide population-based study
Source: Acta Oncol. 2024 Nov 14;63:40137. doi: 10.2340/1651-226X.2024.40137 (PMC11579532; doi:10.2340/1651-226X.2024.40137)
Supplement: The risk of venous thromboembolism in adult patients with diffuse glioma: a nationwide population-based study [file AO-63-40137-s1.pdf]

Supplementary material has been published as submitted. It has not been copyedited, or typeset by Acta Oncologica

Supplementary table 1: Characteristics of patients with glioma WHO grade 2 and 3.

	WHO2				WHO 3			
	**Total n = 230**	**No VTE n = 218**	**VTE n = 12**	**p Value**	**Total n = 317**	**No VTE n = 297**	**VTE n = 20**	**p Value**
**Age at diagnosis (median, range)**	43 (18-87)	43 (18-87)	50 (31-73)	0.23	48 (18-83)	47 (18-83)	59 (30-78)	**0.009**
**Age groups**				0.37				0.13
**18-40**	91 (40%)	87 (40%)	4 (33%)		114 (36%)	110 (37%)	4 (20%)	
**40-60**	99 (43%)	95 (44%)	4 (33%)		125 (39%)	118 (40%)	7 (35%)	
**60-80**	NR	NR	4 (33%)		75 (24%)	66 (22%)	9 (45%)	
**>80**	<3	<3	0 (0%)		3 (1%)	3 (1%)	0 (0%)	
**Sex**				1.00				0.25
**Male**	117 (51%)	111 (51%)	6 (50%)		176 (56%)	162 (55%)	14 (70%)	
**Female**	113 (49%)	107 (49%)	6 (50%)		141 (44%)	135 (45%)	6 (30%)	
**Focal deficit**				0.67				0.95
**Yes**	58 (25%)	NR	<3		100 (32%)	93 (31%)	7 (35%)	
**No**	147 (64%)	139 (64%)	8 (67%)		171 (54%)	161 (54%)	10 (50%)	
**Missing**	25 (11%)	NR	NR		46 (15%)	43 (14%)	3 (15%)	
**Epilepsy**				1.00				0.28
**Yes**	76 (33%)	NR	NR		77 (24%)	NR	<3	
**No**	35 (15%)	NR	<3		47 (15%)	NR	NR	
**Missing**	119 (58%)	113 (52%)	6 (50%)		193 (61%)	179 (60%)	14 (70%)	
**Cognitive change**				0.35				0.73
**Yes**	29 (13%)	26 (12%)	3 (25%)		42 (13%)	NR	<3	
**No**	82 (36%)	79 (36%)	3 (25%)		83 (26%)	NR	NR	
**Missing**	119 (52%)	113 (52%)	6 (50%)		192 (61%)	178 (60%)	14 (70%)	
**Headache**				1.00				0.73
**Yes**	18 (8%)	NR	<3		31 (10%)	NR	<3	
**No**	94 (41%)	NR	NR		94 (30%)	NR	NR	
**Missing**	118 (51%)	112 (51%)	6 (50%)		192 (61%)	178 (60%)	14 (70%)	
**Focality**				0.68				0.80
**Unifocal**	193 (84%)	183 (84%)	10 (83%)		257 (81%)	240 (81%)	17 (85%)	
**Multifocal**	13 (6%)	NR	NR		16 (5%)	16 (5%)	0 (0%)	
**Missing**	24 (10%)	NR	<3		44 (14%)	41 (14%)	3 (15%)	
**Midline crossing**				0.060				0.93
**Yes**	31 (13%)	27 (12%)	4 (33%)		37 (12%)	NR	<3	
**No**	168 (73%)	NR	NR		223 (70%)	NR	NR	

**Missing**	31 (13%)	NR	<3		57 (18%)	53 (18%)	4 (20%)	
**Largest tumor diameter, mm (median, range)**	50 (3-123)	50 (3-123)	60 (40-120)	0.11	47 (4-120)	47 (4-120)	50 (20-80)	0.77
**Charlson Comorbidity Index**				**0.002**				0.73
**0**	104 (45%)	101 (46%)	3 (25%)		63 (20%)	60 (20%)	3 (15%)	
**1-2**	109 (47%)	105 (48%)	4 (33%)		229 (72%)	NR	NR	
**>=3**	17 (7%)	12 (6%)	5 (42%)		25 (8%)	NR	<3	
**IDH status**				0.55				0.58
**Wild type**	0 (0%)	0 (0%)	0 (0%)		0 (0%)	0 (0%)	0 (0%)	
**Mutated**	79 (34%)	74 (34%)	5 (42%)		74 (23%)	71 (24%)	3 (15%)	
**Missing**	151 (66%)	144 (66%)	7 (58%)		243 (77%)	226 (76%)	17 (85%)	
**MGMT status**				0.35				0.92
**Unmethylated**	43 (19%)	NR	<3		32 (10%)	NR	<3	
**Methylated**	31 (13%)	NR	NR		37 (12%)	NR	<3	
**Missing**	156 (68%)	148 (68%)	8 (67%)		248 (78%)	231 (78%)	17 (85%)	
**Type of surgery**				**0.030**				0.56
**Biopsy**	69 (30%)	61 (28%)	8 (67%)		83 (26%)	79 (27%)	4 (20%)	
**Subtotal resection**	73 (32%)	70 (32%)	3 (25%)		119 (38%)	109 (37%)	10 (50%)	
**Maximum safe resection**	NR	NR	NR		115 (36%)	109 (37%)	6 (30%)	
**Missing**	<3	<3	NR		42 (13%)	39 (13%)	3 (15%)	
**Performance status before surgery**				0.42				0.45
**0**	147 (64%)	141 (65%)	6 (50%)		173 (55%)	164 (55%)	9 (45%)	
**1**	48 (21%)	45 (21%)	3 (25%)		65 (21%)	59 (20%)	6 (30%)	
**>=2**	35 (15%)	32 (15%)	3 (25%)		79 (25%)	74 (25%)	5 (25%)	
**Radiotherapy**				**0.035**				0.79
**No**	129 (56%)	126 (58%)	3 (25%)		72 (23%)	67 (23%)	5 (25%)	
**Yes**	101 (44%)	92 (42%)	9 (75%)		245 (77%)	230 (77%)	15 (75%)	
**Chemotherapy**				0.74				1.00
**No**	168 (73%)	160 (73%)	8 (67%)		136 (43%)	127 (43%)	9 (45%)	
**Yes**	62 (27%)	58 (27%)	4 (33%)		181 (57%)	170 (57%)	11 (55%)	

Abbreviations: VTE, venous thromboembolism; IDH, Isocitrate dehydrogenase; MGMT, O^6-methylguanine-DNA methyltransferase promoter.

Supplementary table *2*: Supplementary characteristics of patients with and without VTE for patients with glioma WHO grade 4.

	**Total**<br>n = 3,083	**No VTE**<br>n = 2,875	**VTE**<br>n = 208	**p Value**
**Age groups**				0.22
**18-40**	196 (6%)	188 (7%)	8 (4%)	
**40-60**	922 (30%)	858 (30%)	64 (31%)	
**60-80**	1,766 (57%)	1,639 (57%)	127 (61%)	
**>80**	199 (6%)	190 (7%)	9 (4%)	
**Focal deficit**				0.60
**Yes**	2,037 (66%)	1,906 (66%)	131 (63%)	
**No**	915 (30%)	847 (29%)	68 (33%)	
**Missing**	131 (4%)	122 (4%)	9 (4%)	
**Epilepsy**				0.49
**Yes**	607 (20%)	571 (20%)	36 (17%)	
**No**	1,637 (53%)	1,528 (53%)	109 (52%)	
**Missing**	839 (27%)	776 (27%)	63 (30%)	
**Cognitive deficit**				0.62
**Yes**	1,158 (38%)	1,086 (38%)	72 (35%)	
**No**	1,103 (36%)	1,027 (36%)	76 (37%)	
**Missing**	822 (27%)	762 (27%)	60 (29%)	
**Headache**				0.48
**Yes**	719 (23%)	665 (23%)	54 (26%)	
**No**	1,512 (49%)	1,418 (49%)	94 (45%)	
**Missing**	852 (28%)	792 (28%)	60 (29%)	
**Midline crossing**				0.59
**Yes**	491 (16%)	462 (16%)	29 (14%)	
**No**	2,351 (76%)	2,191 (76%)	160 (77%)	
**Missing**	241 (8%)	222 (8%)	19 (9%)	
**Largest tumor diameter, mm (median, range)**	43 (2-150)	43 (2-150)	43 (3-107)	0.71

Characteristics of patients with glioma WHO grade 4. Continuous measures are presented as median (range) and are compared using the Wilcoxon rank sum test. Categorical measures are presented as n (%) and are compared using the $\chi 2$-squared test or Fisher's exact test if <5 values were observed.

Abbreviations: VTE, Venous thromboembolism.

Supplementary table 3. Distribution of VTE during time intervals before the diagnosis of glioma grade 4.

**Months from glioma diagnosis**	**<-36**	**-36;-24**	**-24;-12**	**-9;-6**	**-6;-3**	**-3;0**	**Total**
**VTE freq. in interval (%)**	41 (56%)	9 (12%)	8 (11%)	4 (5%)	4 (5%)	8 (11%)	74 (100%)
**IR /1000 PY (95%CI)**	1.2 (0.8-1.6)	3.0 (1.5-5.7)	2.6 (1.3-5.3)	1.3 (0.2-9.4)	5.3 (2.0-14.1)	8.0 (3.6-17.7)	1.6 (1.2-2.0)
**Crude VTE incidence**	1.3%	0.3%	0.3%	0.1%	0.1%	0.3%	2.3%

The number of VTE events (%), the incidence rate (95% confidence intervals), and the crude VTE incidence for time intervals before the glioma diagnosis.

Abbreviations: Freq., frequency; IR, incidence rate; PY, person-years.

Supplementary table 4. Distribution of venous thromboembolism during time intervals after the diagnosis of glioma WHO grade 4.

**Months from glioma diagnosis**	**0;3**	**3;6**	**6;9**	**9;12**	**12;24**	**24;36**	**>36**	**Total**
**VTE freq. in interval (%)**	53 (26%)	42 (21%)	34 (17%)	22 (11%)	30 (15%)	10 (5%)	9 (5%)	200 (100%)
**IR /1000 PY (95%CI)**	74.2 (56.7-97.1)	71.7 (53.0-97.0)	71.4 (51.0-99.9)	57.1 (37.6-86.8)	32.9 (23.0-47.0)	23.4 (12.6-43.5)	14.0 (7.3-27.0)	48.3 (42.0-55.5)
**Crude VTE incidence**	1.7%	1.6%	1.6%	1.3%	2.2%	1.7%	3.0	6.5%
**Patients alive**	3,083	2,579	2,107	1,728	1,385	576	304	3,083

The number of VTE events (%), the incidence rate (95% confidence intervals), and the crude VTE incidence for time intervals after the glioma diagnosis as well as the number of patients alive at the beginning of the interval.

Abbreviations: VTE, venous thromboembolism; Freq., frequency; IR, incidence rate; PY, person-years.
